# Applications of Cucurbiturils in Medicinal Chemistry and Chemical Biology

**DOI:** 10.3389/fchem.2019.00619

**Published:** 2019-09-13

**Authors:** Debapratim Das, Khaleel I. Assaf, Werner M. Nau

**Affiliations:** ^1^Department of Chemistry, Indian Institute of Technology Guwahati, Guwahati, India; ^2^Department of Life Sciences and Chemistry, Jacobs University Bremen, Bremen, Germany; ^3^Department of Chemistry, Faculty of Science, Al-Balqa Applied University, Al-Salt, Jordan

**Keywords:** molecular containers, host-guest complexes, drug delivery, supramolecular chemistry, drug release, molecular recognition, chemosensing

## Abstract

The supramolecular chemistry of cucurbit[*n*]urils (CB*n*) has been rapidly developing to encompass diverse medicinal applications, including drug formulation and delivery, controlled drug release, and sensing for bioanalytical purposes. This is made possible by their unique recognition properties and very low cytotoxicity. In this review, we summarize the host-guest complexation of biologically important molecules with CB*n*, and highlight their implementation in medicinal chemistry and chemical biology.

## Introduction

One of the major challenges in modern-day pharmacology and medicine is the stable formulation and targeted delivery of therapeutics (Ghosh and Nau, 2012; Sreenivasolu, 2012; Sanku et al., 2019). A major effort in pharmaceutical research is being invested with the aim to achieve the highest impact of a particular therapeutic agent or drug on living systems by creating appropriate delivery vehicles that affect, on one hand, delivery at the desired target and that protect, on the other hand, drug molecules from degradation. In part, the focus of pharmaceutical research has recently moved more toward the development of new nanoscale biocompatible delivery vehicles and away from the *de-novo* design of new drugs.

Macrocyclic receptors, such as cyclodextrins (CDs), calixarenes (CXs), and cucurbiturils (CBs), have received enormous attention owing to their ability to encapsulate therapeutic agents non-covalently and to release them by appropriate stimuli (Saleh et al., 2013; Liu, 2017). Macrocyclic hosts show considerable advantages over other forms of nano-sized drug carriers (Schneider and Yatsimirsky, 2008). The thermal and chemical stability, formation of different nano-structured assemblies, availability of various sizes, and most importantly, the biocompatibility of these macrocyclic hosts are some of the essential features which differentiate them from alternative drug-delivery vehicles such as dendrimers, liposomes, hydrogels, micelles, carbon nanotubes, or polymers.

Amidst macrocyclic hosts, CDs (Li and Loh, 2008) are the most common choice due to their ready availability, low cost, and high water solubility. However, there are several limitations arising from their poor selectivity and low affinity (*K*_a_ <10^4^ M^−1^) (Rekharsky and Inoue, 1998). Moreover, their use in clinical formulations is generally limited to oral and topical drug delivery because they can be nephrotoxic if administered in non-metabolized form (Shchepotina et al., 2011). The low binding constants, especially toward drug molecules, lead to the requirement of excess concentrations of CDs in order to form host-guest complexes quantitatively.

**Figure 1:**
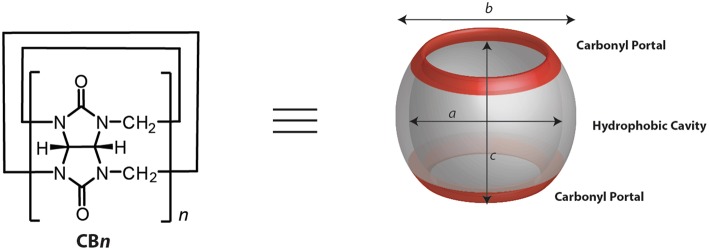
Chemical and model representations of CB*n*.

Several other macrocyclic hosts are under the scanner for development of effective host-guest complexes with drugs in order to stabilize and effectively deliver them. In recent years, CBs (Figure 1) have come out as attractive macrocyclic hosts for applications in medicinal chemistry and chemical biology (Ma and Zhao, 2015; Masson, 2017; Yin and Wang, 2018; Yin et al., 2019). The binding constants (*K*_a_) of their host-guest complexes are several orders of magnitude higher than those of CDs in aqueous medium (Cao et al., 2014; Assaf and Nau, 2015; Barrow et al., 2015; Shetty et al., 2015). Most importantly, CBs hold promise as being non-toxic and highly biocompatible (Montes-Navajas et al., 2009; Hettiarachchi et al., 2010; Uzunova et al., 2010; Zhang et al., 2018b).

CB*n* (*n* = 5–10, 9 yet to be isolated, Figure 1 and Table 1) are readily synthesized from the condensation of glycoluril and formaldehyde in strongly acidic media. Interestingly, though the synthesis was reported back in 1905 by Behrend et al. (1905) the determination of the chemical structure of CB6 took 70 years when Mock and coworkers refined it for the first time crystallographically (Freeman et al., 1981). CB9 is yet to be isolated, but other homologs of CBs (5–10) have in the meantime been purified. Structural analysis of these analogs showed that CBs are macrocycles containing 5 to 10 glycoluril units connected by two methylene bridges on each side of the glycoluril segments. The cyclic structure, thus, creates two identical partially negatively charged hydrophilic carbonyl portals on each sides and a hydrophobic cavity with low polarity and polarizability (Figure 1) (Márquez and Nau, 2001a; Assaf and Nau, 2014).

**Table 1:** Structural parameters^a^ of CB*n* (see Figure 1) and selected physicochemical properties.

***n***	**CB*n***	**Molecular weight**	**Inner diameter *a* [Å]**	**Outer diameter *b* [Å]**	**Height *c* [Å]**	**Inner cavity volume [Å^**3**^]**	**Aqueous solubility *S*_**H2O**_ [mM]**
5	CB5	830	4.4	13.1	9.1	68	20–30^b^
6	CB6	996	5.8	14.4	9.1	142	0.03^c^
7	CB7	1163	7.3	16.0	9.1	242	5^d^
8	CB8	1329	8.8	17.5	9.1	367	<0.01^b^
10	CB10	1661	11.7	20.0	9.1	691	<0.05^e^

a *From Assaf and Nau (2015)*.

b *From Lagona et al. (2005)*.

c *From Márquez et al. (2004b)*.

d *From Márquez et al. (2004a)*.

e *From Liu et al. (2005b)*.

The first two decades on research with CBs were mostly focused on synthesis, structural evaluation, and their guest binding properties. However, with the newer, more economic synthetic and purification strategies, along with considerable knowledge about their properties, the focus has shifted toward applications of this interesting family of water-soluble macrocycles. One of the prominent dimensions of recent publications on CBs is their use in the areas of medicinal chemistry and chemical biology. Though *in vivo* applications of CBs for medicinal and diagnostic purposes are emerging relatively slowly, the increasing number of reports on CB-based drug delivery systems has become overwhelming in the last decade. In this review, we aim to provide an overview of the recent achievements in the area of drug delivery and diagnostics involving host-guest chemistry of CBs. The review focuses on the applications of the parent macrocyclic homologs in medicinal chemistry and chemical biology; applications of acyclic and other variants or derivatives are reviewed elsewhere (Ganapati and Isaacs, 2018).

CBs are well-known to bind a wide range of guest molecules, including small organic molecules, amino acids, peptides, and proteins (Macartney, 2011; Shchepotina et al., 2011; Barrow et al., 2015; Sanku et al., 2019). The association of guest molecules to CBs is generally driven by ion-dipole interactions, as well as the classical and non-classical hydrophobic effect (Nau et al., 2011; Assaf and Nau, 2015). The CB cavity provides a hydrophobic void for the binding of neutral hydrophobic molecules, while the two identical carbonyl rims represent docking sites for positively charged groups, in most cases ammonium groups or other cations. The complexation of hydrophobic residues inside the cavity is associated with the release of high-energy water molecules from the CB cavity, which contributes to the high association constants (Biedermann et al., 2012b, 2014). The size and shape of the guest molecules also modulate the binding process (Nau et al., 2011; Lee et al., 2013; Assaf and Nau, 2015; Assaf et al., 2017). An ideal binding is generally obtained when the guest volume is around 55% of that of the inner cavity of CB*s* (Mecozzi and Rebek, 1998; Nau et al., 2011). Among the CB homologs, CB7 can bind guest molecules with extremely high binding affinities, which exceed that of the biotin-avidin pair, the strongest non-covalent interaction between two partners found in nature (Moghaddam et al., 2011; Cao et al., 2014). The highest binding affinity measured with CBs is 7.2 × 10^17^ M^−1^, observed between CB7 and a diamantane diammonium guest molecule (Cao et al., 2014).

The encapsulation of molecules inside the CB cavity leads usually to (real or apparent) changes in their physical and chemical properties due to an altered microenvironment as well as confinement and isolation from the surrounding medium (Koner and Nau, 2007; Dsouza et al., 2011; Koner et al., 2011). For example, the solubility of poorly soluble drug molecules can be significantly enhanced upon complexation with CBs (Zhao et al., 2008; Koner et al., 2011; Ma et al., 2012a; Lazar et al., 2016). The use of even-numbered CB*n* homologs (*n* = 6 and 8) as drug solubilizing agents is limited due to their low intrinsic solubilities (μM, see Table 1) in water, which can be enhanced to a certain degree in the presence of cations or positively charged guest molecules (Lagona et al., 2005; Masson et al., 2012). Guest molecules can also take advantage of isolation or protection from the bulk solvent upon complexation with CBs. Mohanty et al. reported that CB7 can induce deaggregation and photostabilization of fluorescent dyes, such as Rhodamine 6G, which is commonly used in cell-biological applications such as fluorescence microscopy and fluorescence correlation spectroscopy (Mohanty and Nau, 2005; Nau and Mohanty, 2005). CBs are also known to affect the p*K*_a_ values of the guest molecules and, thereby, alter their chemical reactivities (Koner et al., 2011; Barooah et al., 2012; Ghosh and Nau, 2012; Lazar et al., 2017). The preferential binding of the protonated form of the guest molecule over its neutral form increases the p*K*_a_ values of the conjugate acids of basic guests, leading to complexation-induced p*K*_a_ shifts. The high affinity of the protonated guest is mainly attributed to additional ion-dipole interactions between the cationic sites of the guest molecules with the carbonyl portals of CBs (Márquez et al., 2004b). Also important, the high thermal stability of CBs allows their implementation to improve the thermal stability of many drugs in the solid state (Bardelang et al., 2011; Saleh et al., 2012).

## Encapsulation of Drugs

CB complexation has been established for different classes of drug molecules, pharmaceutical agents, and other bioactive molecules (Hettiarachchi et al., 2010; Huang et al., 2011; Walker et al., 2011; Day and Collins, 2012; Yin et al., 2019). Drug molecules that have been studied for their inclusion complexation with CBs to date include anti-neoplastic, anti-pathogenic, antagonist agents, vitamins and hormones, enzyme inhibitors, neurotransmitters, neuromuscular blockers, anti-tuberculosis agents, local anesthetics, and others. In this section, we provide an overview of the different types of biologically relevant guest molecules in regard to their encapsulation inside CBs.

**Figure 2:**
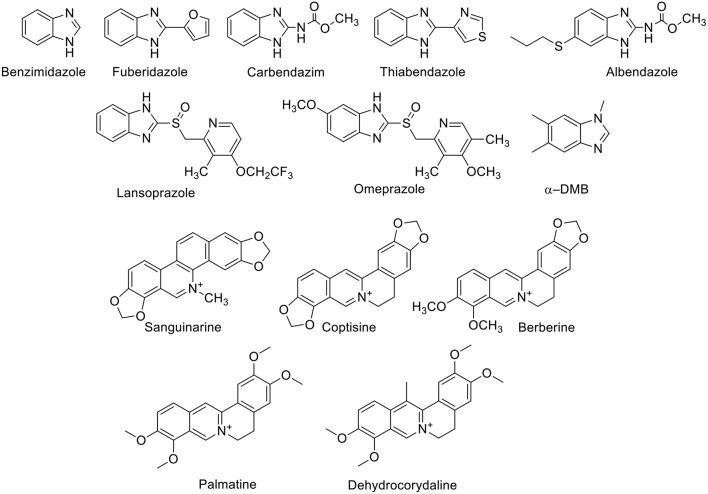
Chemical structures of benzimidazole derivatives and alkaloids which form host-guest complexes with CB*n*.

The supramolecular complexation of benzimidazole-based drugs (Figure 2) has been systematically studied by Nau and coworkers (Saleh et al., 2008; Koner et al., 2011). CB7, in particular, is capable of encapsulating the benzimidazole derivatives albendazole, carbendazim, thiabendazole, and fuberidazole (Saleh et al., 2008; Koner et al., 2011; Tang et al., 2018). These molecules possess very low water solubility in their neutral forms. The p*K*_a_ values of this class of molecules are in the range of 3.5–4.8, and, therefore, they are neutral at physiological pH, which hinders their usability. The binding affinities of benzimidazole derivatives to CB7 in their neutral forms are in the millimolar range; these increase significantly for the protonated forms, reaching micromolar values (Koner et al., 2011). The preferential binding of the protonated forms increases the p*K*_a_ values of the conjugate acids of these drug molecules by 2–5 units and, thereby, improves their solubilities by stabilizing the protonated forms at pH 7.2. For example, CB7 increased the aqueous solubility of albendazole by 2,000-fold (Zhao et al., 2008). Other CB*n* homologs and acyclic derivatives can also enhance the solubility of albendazole (Ma et al., 2012a; Vinciguerra et al., 2012). Beyond the enhanced solubility, CB7 was found to improve the photostability of several benzimidazole drugs (Koner et al., 2011). For example, fuberidazole, and thiabendazole photobleached less effectively in the presence of CB7, with photostabilization factors amounting to 7 and 3, respectively. In addition, CB7 prevents the interconversion of crystal polymorphs of albendazole and retained the amorphous structure in the resulting complex (Saleh et al., 2012).

Sanguinarine (Figure 2), which has anti-oxidant, anti-tumor, anti-bacterial, and anti-inflammatory properties, forms a stable complex with CB7 (Miskolczy et al., 2011). The binding inside CB7 stabilizes the active form of sanguinarine by a complexation-induced p*K*_a_ shift of the alkanolamine from 7.2 to 10.8, allowing its usability in the active form at higher pH values. Further, the complexed sanguinarine was stabilized toward photoirradiation relative to the free drug. CB7 forms a stable host-guest complex with berberine (Figure 2), an antimicrobial agent. The binding was monitored by the fluorescence change of berberine upon complexation, in which the fluorescence of berberine was enhanced by a factor of 500 upon complexation with CB7 (Miskolczy and Biczók, 2014a). With CB8, two berberine units are encapsulated (Miskolczy and Biczók, 2014b). The antimicrobial alkaloid coptisine forms also complexes with CBs, as reflected again in fluorescence changes. The fluorescence intensity of coptisine was greatly enhanced in the presence of CB7, affording a highly sensitive and selective method for the determination of coptisine in aqueous solution (Li et al., 2010). CB7 binds to isoquinoline alkaloids, namely palmatine and dehydrocorydaline (Figure 2) with binding affinities of 2.4 × 10^6^ and 3.2 × 10^4^ M^−1^, respectively (Li et al., 2009). The dramatic fluorescence enhancement upon complexation with CB7 can be observed by naked eye.

**Figure 3:**
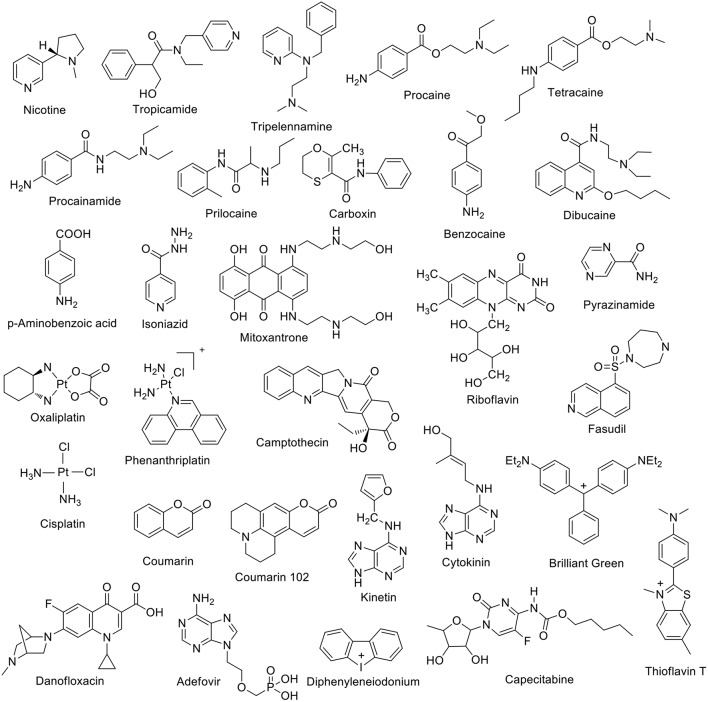
Chemical structures of a first set of selected drug molecules which form host-guest complexes with CB*n*.

Dye displacement was employed to study the complexation of nicotine (Figure 3) by CB7 in aqueous solution (Zhou et al., 2009a). Methylene blue was used as a dye that forms an inclusion complex with CB7 and shows a significant fluorescence response upon complexation. The addition of nicotine, as a competitor guest and analyte, displaced the dye, leading to the restoration of the original dye fluorescence. This allows for the detection of nicotine in concentrations as low as 0.05 μg mL^−1^ (Zhou et al., 2009a). Tropicamide (Figure 3), an antimuscarinic drug routinely applied in eye drops to cause a mydriatic response (pupil dilation) in preparation for ophthalmological examinations and surgery, forms inclusion complexes with CB7 and CB8 in aqueous solution (Saleh et al., 2011). The protonated tropicamide showed high binding affinity with both hosts (*K*_a_ = 1.3 × 10^3^ and 4 × 10^4^ M^−1^ with CB7 and CB8, respectively) (Saleh et al., 2011). Saleh et al. also reported the formation of a host-guest complex between CB7 and the antihistamine drug tripelennamine (Saleh et al., 2016). The binding was studied by means of optical and NMR titrations (Saleh et al., 2016). Macartney and coworkers studied the complexation of local anesthetics with CB7 (Wyman and Macartney, 2010). They found that CB7 can bind procaine (*K*_a_ = 3.5 × 10^4^ M^−1^), tetracaine (*K*_a_ = 1.5 × 10^4^ M^−1^), procainamide (*K*_a_ = 7.8 × 10^4^ M^−1^), dibucaine (*K*_a_ = 1.8 × 10^5^ M^−1^), and prilocaine (*K*_a_ = 2.6 × 10^4^ M^−1^) in acidic aqueous solution. These binding affinities are much higher than those measured for CDs (Wyman and Macartney, 2010). Recently, benzocaine, and its metabolite, *p*-aminobenzoic acid, have been reported to form host-guest complexes with CB7 in water (Li et al., 2016a). The binding affinities are 2.2 × 10^4^ M^−1^ and 1.5 × 10^4^ M^−1^ for the protonated guests, respectively (Li et al., 2016a). The supramolecular interactions of a bactericidal agent against tuberculosis, namely isonicotinic acid hydrazide, commonly known as isoniazid, have been studied with CB6 and CB7 (Cong et al., 2011). The complexation with the macrocyclic hosts hindered the acylation reaction of isonicotinic acid hydrazide (Cong et al., 2011). Complexation of carboxin, a fungicide, with CB8 was found to promote the inhibition activity of carboxin on mycelial growth of *Rhizoctonia solani* (Liu et al., 2011). Relative improvement was evaluated in terms of area covered by the mycelia of *R. solani* and their growth inhibition rate (Liu et al., 2011).

The binding of drug molecules to biomacromolecules can be mediated by the complexation to macrocyclic hosts. For example, the binding affinity of Brilliant Green (BG) to bovine serum albumin (BSA) was enhanced in the presence of CB7 (Bhasikuttan et al., 2007). The CB7 cavity can encapsulate part of the BG molecule, while the unencapsulated part remained accessible to associate to BSA. Mitoxantrone, an anthracenedione antineoplastic agent used to treat certain types of cancer, forms a 2:1 host:guest complex with CB8 (Konda et al., 2017). The complexation increased the mitoxantrone uptake in mouse breast cancer cells and decreased its toxicity (Konda et al., 2017). The complexation of capecitabine with CB7 was investigated by Wang et al. (2018). ITC experiments revealed a 1:1 binding stoichiometry with *K*_a_ = 2.8 × 10^5^ M^−1^. The encapsulation of platinum anticancer drug was reported by Kim and coworkers (Jeon et al., 2005). Oxaliplatin was found to form a 1:1 inclusion complex with CB7 in aqueous solution with a *K*_a_ value of 2.3 × 10^6^ M^−1^. The complexation inside the cavity of CB7 resulted in an enhanced stability (Jeon et al., 2005). The CB7•oxaliplatin complex exhibited cooperatively enhanced antitumor activity compared to oxaliplatin itself (Chen et al., 2017). Phenanthriplatin, an anticancer drug, forms supramolecular complexes with CBs as well (Kahwajy et al., 2017). CB7 accommodates one phenanthriplatin molecule, while the larger cavity of CB8 can simultaneously bind two molecules. The release of phenanthriplatin can be achieved by the addition of cations. NMR studies suggest that cisplatin forms an inclusion complex with CB7, while *cis*-[PtCl(NH_3_)_2_(H_2_O)]^+^ only binds at the portals (Wheate et al., 2006). The formation of a 1:1 riboflavin•CB7 complex in aqueous solution (*K*_a_ = 1.25 × 10^4^ M^−1^) has also been reported (Zhou et al., 2009b). Coumarin, an anti-coagulent, was found to form stable inclusion complexes with CB7 and CB8 in aqueous solution (Wang et al., 2009). The binding constant with CB7 was measured as 2.6 × 10^5^ M^−1^. Crystal structures revealed the encapsulation of two coumarin units inside CB8 (Wang et al., 2009). Fasudil (FSD), a *roh* kinase inhibitor, forms a stable supramolecular host-guest inclusion complex with CB7 with a binding constant of *K*_a_ = 4.28 × 10^6^ M^−1^ under acidic conditions (pH = 2.0) (Yin et al., 2017). The effect of camptothecin complexation with CB*n* (*n* = 7 and 8) on its solubility and reactivity as an anticancer drug was reported by Dong et al. (2008). The solubility of camptothecin was enhanced up to 70 and 8 times at pH 2 due to the formation of host-guest complexes with CB7 and CB8, respectively. Further, the formed host-guest complexes retained the characteristic camptothecin activity (Dong et al., 2008). Kinetin (Figure 3), a plant hormone that promotes cell division, forms inclusion complexes with CB7 and substituted CB6 derivatives in aqueous solution as well as in the solid state (Huang et al., 2008b). A magnetic perhydroxy-CB8 material was prepared that showed good adsorption capacity for cytokinins (Zhang et al., 2016). CB7 was reported to form stable complexes with the H2-receptor antagonist ranitidine, the administration of which is one of the most popular treatments of stomach ulcer symptoms (Wang and Macartney, 2008). The stability of the ranitidine complexes varies for the diprotonated (*K*_a1_ = 1.8 × 10^8^ M^−1^), monoprotonated (*K*_a2_ = 1.0 × 10^7^ M^−1^), and neutral form (*K*_a3_ = 1.2 × 10^3^ M^−1^). The CB7 complex was also found to improve the thermal stability of the drug (Wang and Macartney, 2008).

The complexation of diphenyleneiodonium (Figure 3), a bioactive halonium ion, with CB7 and CB8 has been recently reported (Yin et al., 2018). Host-guest binding experiments revealed a 1:1 complexation stoichiometry with CB7 (*K*_a_ = 3 × 10^4^ M^−1^) and a 1:2 one with CB8 (*K*_a_ = 2 × 10^12^ M^−1^). Interestingly, the complexation was shown to modulate the inhibitory activity of diphenyleneiodonium against reactive oxygen species generation and to alleviate its cardiotoxicity.

Recently, the complexation of a third-generation fluoroquinone, danofloxacin (Figure 3), by CB7 has been investigated (El-Sheshtawy et al., 2018). The complex was found to be stable at different pH values (*K*_a_ = 10^3^-10^5^ M^−1^). The antibacterial activity of danofloxacin, and two additional second-generation fluoroquinones, i.e., norfloxacin and ofloxacin, was enhanced in the presence of CB7. Feng et al. studied the interaction between CB7 and the hepatitis B drug Adefovir (Figure 3) (Feng et al., 2019). Adefovir forms a 1:1 complex with CB7 with *K*_a_ = 4.25 × 10^3^ M^−1^. The thermal stability of Adefovir was enhanced upon complexation.

**Figure 4:**
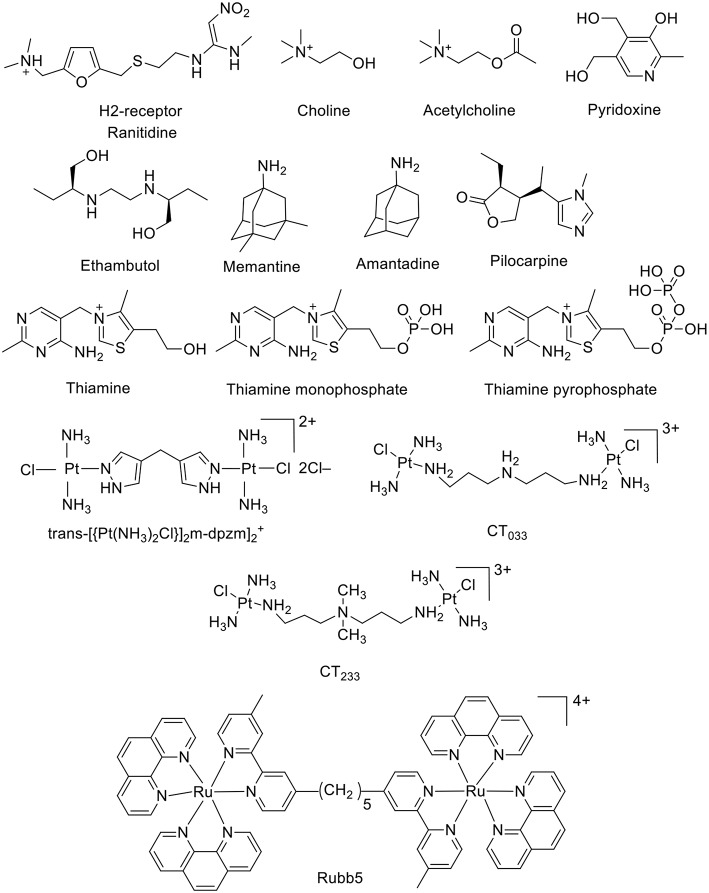
Chemical structures of a second set of selected drug-based molecules which form host-guest complexes with CB*n*.

The CB6 derivative (allyloxy)_12_CB6 forms a stable supramolecular host-guest complex with acetylcholine (*K*_a_ = 5 × 10^3^ M^−1^, Figure 4) and a much weaker complex with choline (Kim et al., 2012; Ghale and Nau, 2014). An indicator displacement strategy was developed for the detection of ethambutol (Figure 4) in water as well as in biological fluids (Wu et al., 2011). The complexation of ethambutol to CB7 was observed upon the release of the precomplexed fluorescent dye (Wu et al., 2011). Adamantane derivatives have found practical application as drugs. The hydrophobic nature of the adamantane residue is well-known in the CB field as a gold-standard with high-binding affinity (Liu et al., 2005a; Assaf and Nau, 2015). For example, amantadine and memantine form exceptionally stable complexes with CB*n* (*n* = 7 and 8) (Vázquez et al., 2014; Assaf and Nau, 2015). Pyridoxine, also known as vitamin B6 or pyridoxol, could be encapsulated inside the CB7 cavity in aqueous solution (*K*_a_ = 4.0 × 10^3^ M^−1^) (Li et al., 2016d). The 1:1 complexation pattern was characterized by ^1^H NMR and UV-Visible spectroscopy (Li et al., 2016d). The interaction between the CB7 macrocycle and pilocarpine was investigated in aqueous solution by using ^1^H NMR and circular dichroism spectroscopic techniques (Saleh et al., 2014). The protection of the lactone group showed a significant enhancement upon the chemical stability of pilocarpine against hydrolysis in basic aqueous solution (Saleh et al., 2014). Thiamine, thiamine monophosphate, and thiamine pyrophosphate form 1:1 host-guest complexes with CB7 as well (Li et al., 2016b). The host–guest stability constants were determined by UV-Visible titrations. The presence of an anionic phosphate/diphosphate group on the molecular structures lowered the binding affinity (Li et al., 2016b).

Collins and Day investigated the interactions of the antibiotic drugs *trans*-[(PtCl(NH_3_)_2_)_2_(μ-NH_2_(CH_2_)_8_NH_2_)]^2+^ and [(Ru(phen)_2_)_2_(m-bb_5_)]^4+^ {phen = 1,10-phenanthroline; bb_5_ = 1,5-bis[4(4'-methyl-2,2'-bipyridyl)]-pentane) (Rubb5, Figure 4) with CB macrocycles. ^1^H NMR experiments indicated that the platinum group at both ends of the *trans*-[(PtCl(NH_3_)_2_)_2_(μ-NH_2_(CH_2_)_8_NH_2_)]^2+^ were too large to allow the threading through the portal of CB6. On other hand, CB7 and CB8 were able to bind the platinum complex, in which all methylene groups are located inside the cavity, while the platinum centers docked at the CB portals (Pisani et al., 2010). The complexation with CBs prevented the degradation by biological nucleophiles. The large cavity of CB10 could also serve as a delivery vehicle for these potential drugs (Pisani et al., 2010; Deng et al., 2018).

**Figure 5:**
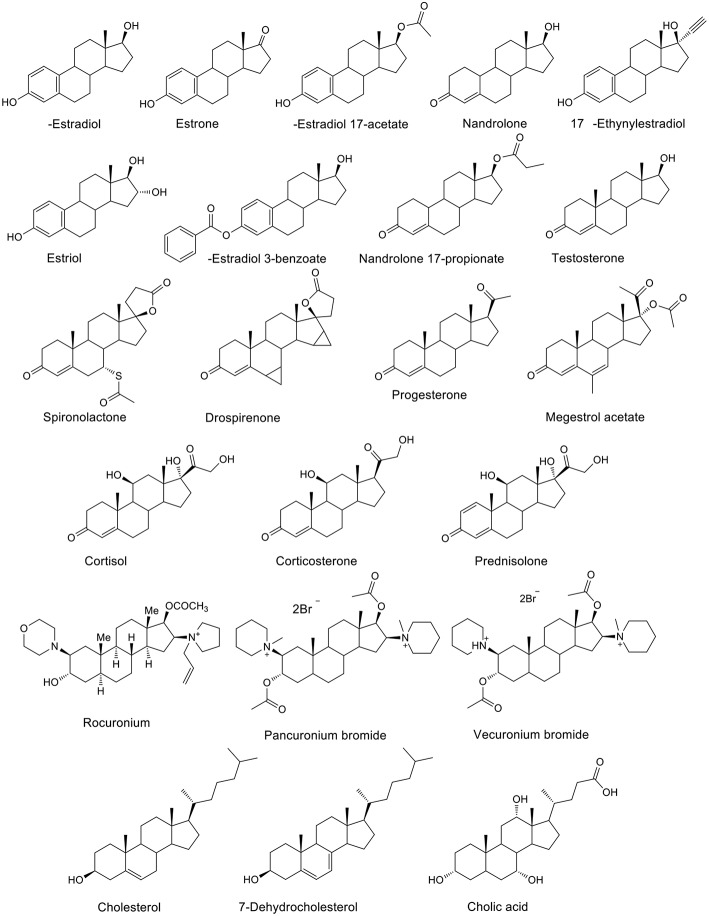
Chemical structures of steroids which form host-guest complexes with CB7 and CB8.

CB*n* (*n* = 7 and 8) act as artificial organic receptors for steroids (Figure 5), including the hormones testosterone and estradiol, the inflammation inhibitor cortisol, as well as the muscle relaxants pancuronium and vercuronium, with extraordinarily high binding affinities (Lazar et al., 2016). For example, CB8 binds preferentially estranes, androstanes, and pregnanes, while CB7 binds nandrolone selectively. The high affinities are also retained in buffered water as well as in biological media such as gastric acid and blood serum. Three steroidal neuromuscular blocking agents, rocuronium, vecuronium, and pancuronium have been investigated as candidate guest molecules for CB7. In aqueous solution, CB7 binds the steroidal neuromuscular blockers with high affinity, following the order: vecuronium > pancuronium > rocuronium (Gamal-Eldin and Macartney, 2014).

CB*s* can selectively accommodate and interact with amino acids and small peptides in water (Bush et al., 2005; Urbach and Ramalingam, 2011; Gamal-Eldin and Macartney, 2013; Biedermann and Nau, 2014; Lee et al., 2015; Smith et al., 2015; Kovalenko et al., 2016; Bai et al., 2017). The binding of amino acids and their corresponding decarboxylated adducts to CB7 was explored by Bailey et al. The study revealed a higher affinity for the decarboxylated molecules (Bailey et al., 2008). Urbach and coworkers showed that the binary CB8•methyl viologen complex can selectively bind peptides with N-terminal tryptophan compared to C-terminal or internal tryptophan residues through the formation of a ternary complex (Bush et al., 2005). Recently, selective peptide recognition has also been documented for methionine-terminated peptides with CB8 as a receptor without any auxiliary guest (Hirani et al., 2018). The binding of human insulin by CB7 *in vitro* was also reported (Chinai et al., 2011). Its recognition relies on the binding of N-terminal phenylalanine to CB7 (*K*_a_ = 1.5 × 10^6^ M^−1^) (Chinai et al., 2011).

## Mechanisms of Drug Release from Cucurbituril-Based Systems

**Figure 6:**
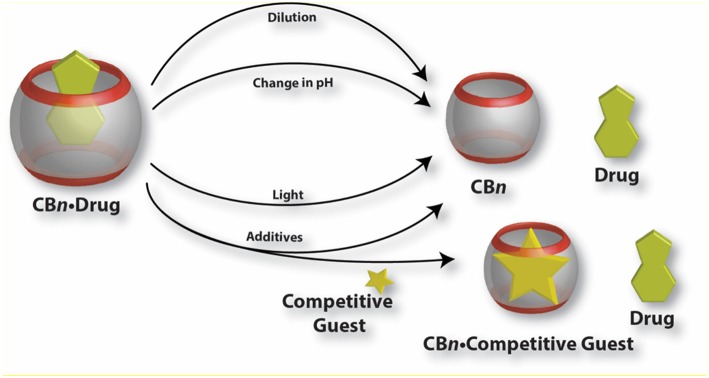
Graphical presentation of various mechanisms for the release of drug molecules from CB•drug complexes.

A schematic presentation of various ways to release encapsulated drugs from CB•drug complexes is shown in Figure 6.

### Dilution Effect

Dissociation of CB•drug complexes to release the drug molecules in general follows a fast kinetics and the association and dissociation rate constants fall in the order of seconds or faster which ensures a fast dynamic equilibrium for rapid drug release. However, very slow release with dissociation rate constants in the range of hours has also been reported, potentially suitable for sustained release. Albendazole (Figure 2), an antiparasitic agent, was found to be released within seconds from CB7, while the release of dinuclear ruthenium complexes (Figure 4) from the cavity of CB10 takes several hours (Zhao et al., 2008; Pisani et al., 2010). One important factor controlling the dissociation is the dilution, which inevitably occurs when a CB•drug complex enters the body fluid. Complexes with macrocycles are held together by weak non-covalent forces, which can be disrupted after administration, such that the CB•drug complexes encounter a lowering in concentration. Invariably, dilution decreases the degree of complexation. Thus, the release of the administered drug will spontaneously occur simply because of the associated dilution effect. It is worth mentioning that an accelerated release of drugs is not always desirable; for certain treatments, a sustained and slow release may be preferred to achieve the highest therapeutic effect. In the case of *cis*-platin (Figure 3), as reported by Wheate and coworkers, the encapsulated drug inside CB7 showed a much slower release rate *in vivo* than in the *in vitro* experiments (Plumb et al., 2012). A direct consequence is the retention of complexed *cis*-platin in circulation for a longer time than of the free drug which leads to better efficacy of the drug. In this regard, one also needs to consider that the absolute binding affinities of the guest molecules with CBs are not always a useful measure of the kinetics of drug release; the tight carbonyl portals of CBs may present a steric/mechanical barrier toward ingression and egression of larger guests, a phenomenon known as constrictive binding (Márquez and Nau, 2001b; Márquez et al., 2004b; Pisani et al., 2010). Though the dynamic complexation-decomplexation of the drug molecules from the CB cavity is found effective in certain cases, it is equally important to incorporate stimuli responsiveness to the complexes which may lead to the release of drugs at a specific location, time, or rate.

### Effect of Additives

**Figure 7:**
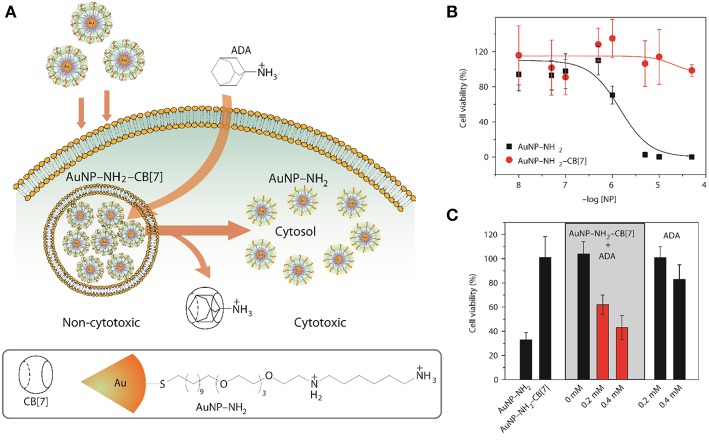
**(A)** Schematic illustration for the use of intracellular supramolecular host–guest complexation to trigger nanoparticle cytotoxicity; **(B,C)** Cytotoxicity of AuNP–NH_2_ and AuNP–NH_2_-CB7 and modulation of cytotoxicity of the gold nanoparticles (Kim et al., 2010a) (Reproduced with permission, Copyright 2010, Nature publishing group).

Another viable way of releasing encapsulated drugs is the use of inorganic cations. Inorganic cations competitively displace cationic guests (Shaikh et al., 2008) from the CB cavity by binding at the portals. As a consequence, the effective binding constants of the guests always get reduced in the presence of salts (Márquez et al., 2004b; Bhasikuttan et al., 2011). Importantly, biological fluids naturally contain large amounts of salts which can trigger the release of drugs from CB complexes. As a proof of concept, it was shown that salts can shift the equilibrium from CB7-bound methyl red to the dye bound in the hydrophobic pocket of BSA (Shaikh et al., 2008). Shaikh et al. have shown that the 1:1 and 1:2 complexes of Thioflavin T (ThT, Figure 3) and CB7 respond differently to the presence of salts. In case of the 1:1 complex, the consequence of addition of salts is the release of the dye while in case of the 1:2 complex, metal ions result in the formation of a capsule-like structure (Choudhury et al., 2009, 2010). The addition of a competitive guest in the form of 1-adamantylamine (ADA), an antiviral and antiparkinsonian drug itself, leads to the destruction of the capsular complex. A competitive guest can also be effective in releasing the drug molecules from the CB cavity. Kim et al. demonstrated that CB7-stabilized amine-functionalized gold nanoparticles (AuNP-NH_2_) can be ruptured by ADA to release AuNP-NH_2_ (Figure 7) and, thereby, enhance their cytotoxicity toward MCF-7 cells (Kim et al., 2010a).

The release of entrapped drugs from self-assembled systems can also be achieved by addition of macrocycles other than the ones used to form the self-assembly (Wu et al., 2016). The affinity of two macrocycles for different regions of the drug can be used to disassemble the system. As demonstrated by Wu et al., noncovalent association of alkyl-chain modified polyamines with CB6 decreased the critical aggregation concentration significantly and led to the formation of self-aggregated nanoparticles (Wu et al., 2016). CDs, which have a higher binding affinity to the hydrophobic chain, disrupt these doxorubicin-(DOX)-loaded nanoparticles to release the drug molecules. The DOX-loaded nanoassembly exhibited better anticancer activity toward MCF-7 cancer cells, but was safe to normal cells. Singharoy et al. showed that the release of a naphthalimide derivative, [2-(2-aminoethyl)-1H-benzo[deisoquinoline-1,3(2H)-dione], from the cavity of CB7 can be modulated by the addition of surfactants (Singharoy et al., 2017). In the presence of non-ionic surfactants, e.g., Ig-720, the drug can be effectively released from CB7, while ionic surfactants were less effective (Singharoy et al., 2017).

### Changes in pH

The inclusion of protonated guests by macrocyclic hosts often results in a shift in the p*K*_a_ values of the guests (Márquez and Nau, 2001b; Saleh et al., 2008). The observed direction of the shift depends on the host and its inclination for binding with the protonated guest compared to its conjugate base. In case of CBs, in general, the p*K*_*a*_ values of basic guests increase as they are encapsulated inside the CB cavity. The switch in the p*K*_*a*_value can be of great importance for the release of the guest molecule. A subtle change in pH of the system can lead to the decomplexation of the CB•drug complexes. A pH jump of the medium from below the p*K*_a_' (the p*K*_a_ value of the complex) to above effectively reduces the binding constant of the drug and affects its fast release through changes of the chemical equilibrium toward the uncomplexed guest (and host).

**Figure 8:**
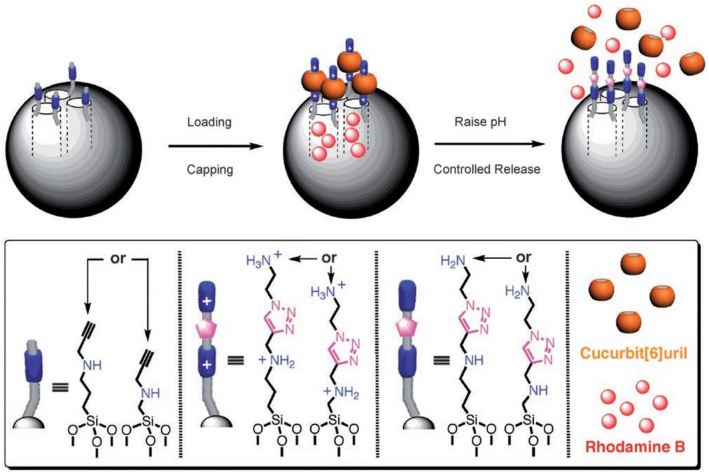
Graphical representations of operational supramolecular nanovalves (Angelos et al., 2008) (Reproduced with permission, Copyright 2008, Wiley-VCH Verlag GmbH and Co. KGaA, Weinheim).

A pH-responsive drug release was demonstrated by Zink and Stoddart in the form of surface-immobilized pseudorotaxane-based nanovalves (Figure 8) (Angelos et al., 2008, 2009). Mesoporous silica nanoparticles functionalized with alkyne groups were loaded with Rhodamine B and, subsequently, the surface was functionalized by means of an interfacial CB6-catalyzed 1,3-dipolar cycloaddition of the alkyne groups and 2-azidoethylamine. This resulted in the formation of CB6/disubstituted 1,2,3-triazole[2]pseudorotaxanes which acted as nanovalves. An increase in pH of the system leads to the opening of nanovalves as the inclusion complex breaks and consequently, the loaded dye gets released. The pH-dependent binding of CB6 with the bisammonium stalks presents the operational principle of these nanovalves.

### Light-Triggered Release of Drugs From Cucurbituril•Drug Complexes

**Figure 9:**
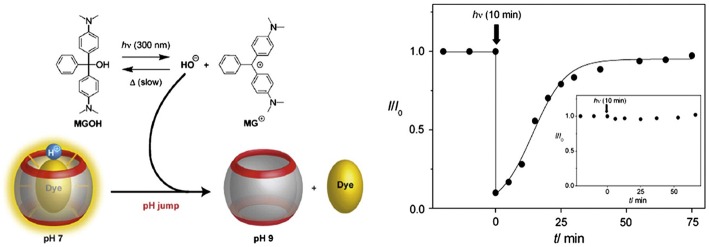
Graphical representation of a photo-triggered pH jump-induced release of an encapsulated dye (MGOH, malachite green leuco hydroxide) from the cavity of CB7 (Carvalho et al., 2011) (Reproduced with permission, Copyright 2011, The Royal Society of Chemistry).

A photo-triggered change in pH and associated release of the guest from the complexed form is also possible, as demonstrated by Carvalho et al. (2011). In this model, the authors used Hoechst 33258 as a guest for CB7 and malachite green leuco hydroxide (MGOH) as a photo-base. The binding constant of protonated Hoechst 33258 with CB7 is 100 times higher than that of the neutral form of the dye. Upon irradiation with UV light, MGOH generates OH^−^ and increases the pH of the solution from 7 to 9. This stimulus affects the release of Hoechst 33258 from the host cavity (Figure 9). A negative control in buffer resulted in no release of the drug. Photo-induced release of drugs can also be materialized by using appropriate photo-responsive molecules. Basílio and Pischel described the photo-controlled release of a widely used Alzheimer's drug, 3,5-dimethyl-1-aminoadamantane, also known as memantine, based on the photo-induced transformation of chalcone to flavylium (Basílio and Pischel, 2016). Charged flavylium can be generated by irradiating non-charged chalcone (three orders of magnitude lower affinity to CB7 than flavylium), which can effectively release the drug from its CB7•drug complex. Recently, Romero et al. reported a light-induced release of a tripeptide from the cavity of CB8 by employing the chalcone/flavylium photo-switch in conjunction with light and acid as input signals (Romero et al., 2018). The flavylium cation, which resulted from a pH-dependent and light-induced transformation of chalcone upon irradiation at 365 nm, acts as a competitive binder for CB8 and, thus, triggers the release of the tripeptide from the cavity (Romero et al., 2018).

The concept of nanovalves on mesoporous silica nanoparticles (Figure 8) was further extended to the controlled release of entrapped guests by light through a photothermal mechanism involving the plasmonic properties of a gold nanoparticle core (Croissant and Zink, 2012). For the preloaded guests (inside the pores) of a mesoporous silica matrix containing embedded gold nanoparticles, the release of the guest molecule could be triggered by laser irradiation. Laser irradiation with low intensity at the wavelength corresponding to the plasmon resonance of the gold nanoparticles causes a local internal heating through dissipation of the photonic energy, which raises the local temperature above 60°C to significantly decrease the ring-stalk binding and, thus, release the guest molecules. This light-sensitive nanostructure can increase the local temperature without significantly changing the bulk temperature, which could potentially be used for (spatially) controlled dual therapy involving the delivery of drug molecules to cells and necrosis through hyperthermia.

### Release From Micro-Heterogeneous Systems

Apart from inclusion complexation-based drug release, CBs were successfully implemented in constructing micro-heterogeneous systems which can entrap and release drug molecules. Construction of supramolecular peptide-amphiphiles using ternary complexation presents one example (Jiao et al., 2012; Mondal et al., 2015). Supramolecular peptide amphiphiles (SPAs) and their vesicle formation were reported where the SPAs were prepared with a viologen amphiphile and peptides containing an appropriate second guest. In their pioneering work, Scherman and coworkers have shown that the vesicles formed by the SPAs were taken up by HeLa cells and responded to multiple external triggers, which could modulate the toxicity of the supramolecular system (Jiao et al., 2012).

Hydrogels are another class of materials, which are being considered as potential targeted drug delivery vehicles. CB6-containing alginate hydrogel beads were found to load an anti-cancer drug, 5-fluorocil (FU), with a loading capacity of 3.87–6.13 wt%. These drugs can then be slowly released. The optimal (slowest) release, with a half-life of 2.7 h, was found for a loading of 5.94% (Huang et al., 2008a).

Nano-assemblies of CBs with proteins were also used as an effective way for the construction of stimuli-responsive materials for controlled release of drugs. A hybrid of bovine serum albumin (BSA) and CB7 formed a non-toxic nano-assembly which can load an anti-cancer drug, DOX and effectively release it in the presence of ADA or a change in pH. Importantly, the dis-assembly of the composite led to restoration of the BSA structure and its recognition property. The DOX-loaded assembly was observed to mask the cytotoxicity of DOX and the toxicity can be restored at the target on demand, triggering its therapeutic activity (Barooah et al., 2017).

Another important type of nanoscale assemblies are supramolecular polymers (Yang et al., 2015). In recent years, a considerable number of such supramolecular polymers have been reported that are based on CBs (Appel et al., 2012; Stoffelen et al., 2015; Ahmed et al., 2016). Loh et al. reported a micelle-like structure formed by supramolecular assembly of poly(N-isopropylacrylamide (as a thermoresponsive block) and poly(dimethylamino-ethylmethacrylate) (as the pH-responsive block) (Loh et al., 2012). These two blocks are held together by ternary complexation of CB8. DOX was encapsulated inside this micelle-like structure and intracellular delivery of the drug was demonstrated using three stimuli, namely, pH, temperature, and a competitive binder. The micellar structure disrupted upon changing the pH from 7 to 4, upon lowering the temperature from 37°C of 15°C, and upon addition of ADA. The release of DOX from the micellar core to the nuclei of HeLa cells was also observed within a desirable time frame.

**Figure 10:**
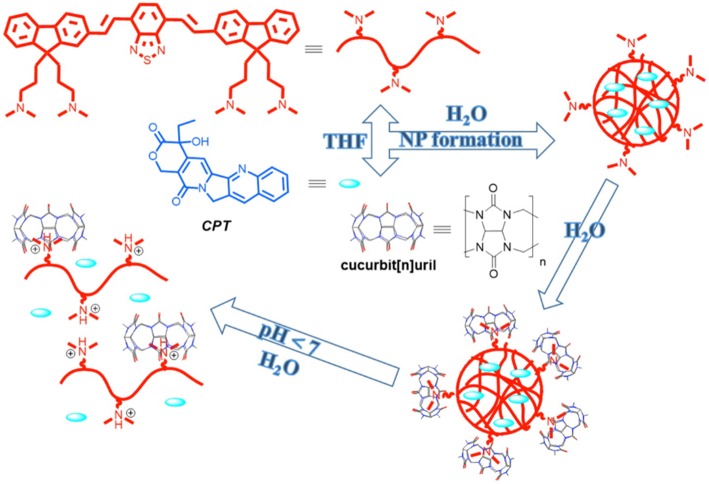
Preparation of CB-capped drug-loaded nanoparticles (NP) and illustration of a pH-triggered drug release mechanism (Pennakalathil et al., 2014) (Reproduced with permission, Copyright 2014, American Chemical Society).

In a recent study, Tuncel and coworkers reported the synthesis of nanoparticles based on a conjugated oligomer (Pennakalathil et al., 2014). The nanoparticles could carry camptothecin, an anticancer drug, with high loading efficiency. The cell viability studies with breast cancer cell lines showed that the *IC*_50_ values of the nanoparticles for MCF7 and MDA-MB-231 were 44.7 and 24.8 μM, respectively. The cytotoxicity of the nanoparticles was further decreased by capping the amine groups with CB7. *IC*_50_ values for camptothecin in the presence of nanoparticles with or without CB7 were significantly reduced in MCF7 and MDAMB-231 cells. CB7-capped drug-loaded nanoparticles regulated the release rate by providing much slower release at pH 7.4 than the nanoparticles in the absence of CB7 (Figure 10).

**Figure 11:**
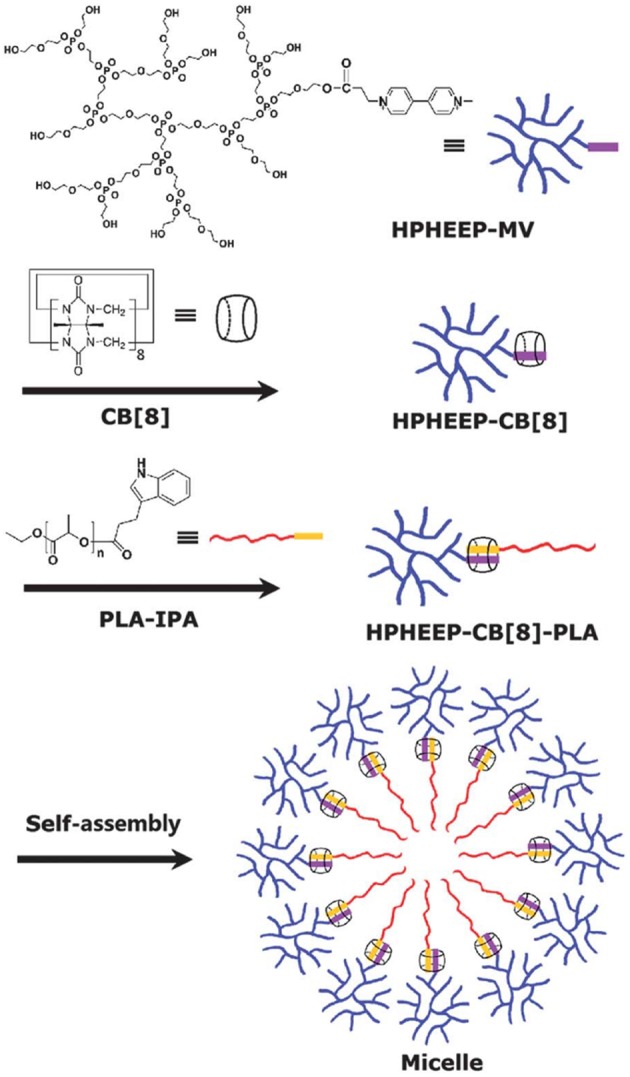
Methyl viologen-functionalized hyperbranched polyphosphate (HPHEEP-MV) and indole-terminated poly(D,L-lactide) (PLA-IPA) assembly via ternary complexation inside CB8 (Chen et al., 2013) (Reproduced with permission, Copyright 2013, The Royal Society of Chemistry).

Alternatively, a redox trigger can be applied to release entrapped drug molecules from polymeric materials. Methyl viologen-(MV)-functionalized hyperbranched polyphosphate (HPHEEP-MV) and indole-terminated poly(D,L-lactide) (PLA-IPA) can be conjugated via ternary complexation inside CB8 (Figure 11) (Chen et al., 2013). The amphiphilic ternary complex could form micelles where HPHEEP remains at the surface while the interior is made of PLA. The micelles could be disrupted by the addition of ADA or Na_2_S_2_O_4_ through competitive binding or formation of radical cations of MV, respectively. The disruption of the micellar structure results in the release of the loaded hydrophobic drug Coumarin 102 (Figure 3). In another example, the team has reported a micellar assembly via ternary complexation of viologen-functionalized poly(ethylene oxide) (PEO) and PLAIPA (Zhao et al., 2014). The micelles were loaded with DOX and the release of the drug could be triggered by reduction with Na_2_S_2_O_4_. *In vitro* cell viability studies indicated good biocompatibility of the micelles toward two cell lines, that is, human umbilical vein endothelial cells (HUVEC) and human liver cancer HepG2 cells. Enhanced toxicity was observed.

## Targeted Drug Delivery from Cucurbituril-Based Systems

In the previous section, we have discussed the different mechanisms which can be used to release encapsulated drugs from either inside CBs or CB-based self-assemblies. However, the bigger challenge is to create the “magic bullet” which can specifically target the diseased cell and deliver the therapeutic site-selectively. Researchers have recently concentrated their efforts on preparing new CB-based systems which can deliver drugs at the targeted site. The present section encompasses a summary for most of the reports on such CB-based targeted drug delivery systems.

Targeting can be achieved via appropriate functionalization of the carrier system with functional groups that are recognized by specific receptors present at the cell surface. Incorporation of these functional groups into the system can either be achieved by covalent or non-covalent attachment of such groups to the self-assembled delivery vehicle. A promising example is the synthesis of a functionalized CB6 derivative that assembles into vesicles (Lee et al., 2005). The surface of the vesicles can be decorated via non-covalent interactions of alkylammonium-tagged guests with free CB6 cavities. When the surface of the vesicles was decorated with a thiourea-linked α-mannose-spermidine conjugate and mixed with a solution containing Concanavalin A (ConA), a lectin that shows specificity toward α-mannose, immediate aggregation was observed. The use of a galactose derivative instead of mannose did not show any aggregation. The resulting system can be potentially applied to diseases where mannose receptors are over-expressed.

Functionalized CB6 was also used to prepare nanoparticles loaded with Nile red (NR, as a model hydrophobic drug) and decorated with spermidine-conjugated folate via host-guest chemistry of CB6 and spermidine (Park et al., 2009). The folate-decorated system showed effective uptake of the dye into HeLa cells whose surface has overexpressed folate receptors. A negative control with nanoparticles lacking folate resulted in no or minimal uptake of the dye. Folate receptor-mediated endocytosis was confirmed as uptake mechanism. After endocytosis, Nile red was released, as monitored by confocal laser scanning microscopy. Building on these findings, the unloading of the antitumor drug, PTX, to HeLa cells was also established. A galactose-functionalized CB6-based carbohydrate wheel was also used to demonstrate galactose-receptor mediated endocytosis into HepG2 hepatocellular carcinoma cells (Kim et al., 2007). In a complementary study, the same CB6-galactose conjugate was utilized to non-covalently encapsulate dextran-spermine conjugates into hepatocyte cells containing asialoglycoprotein (ASGPR) receptors. This model was also used to demonstrate the viability of a non-toxic and biocompatible receptor-mediated gene delivery system (Kim et al., 2010c).

A polymeric nanocapsule consisting of a disulfide-bridged CB6 was reported by Kim et al. Treatment with dithiothreitol (DDT), a reducing agent, breaks the disulfide linkage and ruptures the nanocapsule to release the pre-loaded dye (Kim et al., 2010b). The potential application of this system in targeted drug delivery was illustrated by encapsulating a galactose-spermidine conjugate into the CB cavity and, thereby, bringing the galactose moiety to the surface of the nanocapsules. Carboxyfluorescein was used as an imaging probe. After incubation with HepG2 hepatocellular carcinoma cells, a change in fluorescence inside the cells was observed, indicating the cellular uptake of the entire system. Controlled *in vitro* targeted release of DOX in HeLa cells has also been reported according to the same principle (Park et al., 2010).

**Figure 12:**
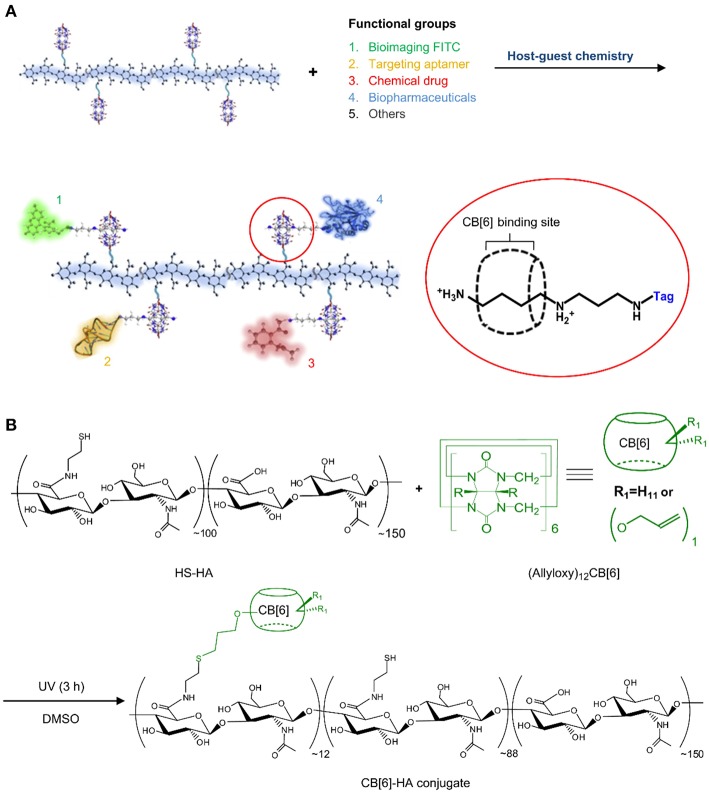
**(A)** Schematic illustration of a multi-functional theranostic system using CB6-HA tethered with various functional “tag”-spermidine conjugates by the host-guest complexation between spermidine and CB6. **(B)** Preparation of a CB6-HA conjugate by UV photoreaction of thiolated hyaluronate (HS-HA) with (allyloxy)_12_CB6. FITC, fluorescein isothiocyanate (Jung et al., 2011) (Reproduced with permission, Copyright 2013, Elsevier).

In another work, CB6-conjugated hyaluronate (CB6-HA) was synthesized and non-covalently decorated with a peptide-spermidine conjugate (Jung et al., 2011). The peptide-spermidine was used as a model for a drug that binds to and activates the formyl peptide receptor (FPRL1). A FITC-spermidine conjugate was used as imaging probe (Figure 12). Controlled drug targeting into B16F1 cells with HA receptors was confirmed *in vitro* by simultaneous bioimaging of FITC-spermidine-conjugated CB6-HA. Activation of the FPRL1 receptor results in enhanced intracellular Ca^2+^ levels, through which the delivery of the CB6-HA-peptide-spermidine conjugate could be demonstrated in FPRL1-expressing human breast adenocarcinoma (FPRL1/MCF-7) cells. The bright fluorescence signal of FLUO-3/AM served as indicator for enhanced Ca^2+^ concentrations. The stability of the system in biological media was also demonstrated *in vitro* as well as *in vivo*.

Recently, a CB7-PEG copolymer was developed as drug carrier. The accessible cavity of CB7 was able to encapsulate the anticancer drug oxaliplatin. The supramolecular polymeric material displayed low cytotoxicity to normal cells. However, the cytotoxicity of the encapsulated oxaliplatin was recovered in cancer cells. The release of the anticancer drug is attributed to the high concentration of spermine in cancer cells, which acts as a competitive guest and, thereby, trigger the release of the complexed drug in a targeted manner (Chen et al., 2018).

Zhang and coworkers selected MV as model antitumor agent and demonstrated an elegant targeted delivery application, which is also based on overexpressed spermine (Chen et al., 2016). MV is highly toxic in nature and affects both tumor and normal cells without specificity. When encapsulated in CB7, the cytotoxicity of MV to normal cells decreased significantly. However, for tumor cells, the overexpressed spermine displaces the encapsulated MV from the complex, thereby allowing the recovery of cytotoxicity of MV.

## Cucurbituril•Drug Complexes in Pharmaceutical Formulations

As discussed before, the stability of CB•drug complexes depends on the medium and presence of other components in the system. Similarly, the property and, hence, the preferred administration mode also depends on the biological media. The presence of salt and acid in biological media affect the solubility of CBs significantly (Steed and Gale, 2012). For example, the solubility of CB6 in simulated gastric fluid increases up to 4 mM (Walker et al., 2010) compared to 0.03 mM (Márquez et al., 2004a) in water. Moreover, the different solubilities of the members of the CB family may dictate the most promising way of administration of the CB•drug complexes (Steed and Gale, 2012; Venkataramanan et al., 2012; Saleh et al., 2013).

The production of CB-based host-guest complexes as solid products involves mixing of the hosts and guests in appropriate stoichiometry, isolation of the complexes in solid form using either lyophilization (Zhao et al., 2008), co-solvent processing (Blanch et al., 2002), or ball-mill grinding (Constabel and Geckeler, 2004; Jiang and Li, 2006; Walker et al., 2010). A fundamental issue which needs to be taken care of is to ensure that the components are held together by non-covalent interactions. To shift the dynamic chemical equilibrium toward the bound drug in solution, the concentration of the host needs to be adjusted such that the drugs are mostly present in their complexed forms. As an asset, the guest binding affinity of CBs is exceptionally high which facilitates the preparation of solutions with 99% or even higher content of the complexed drug even at relatively low (excess) CB concentrations.

The simplest, safest, most convenient, and most common drug administration way is the oral route. Thus, formulation and production of CB-drug complexes in the form of tablets is essential (Walker et al., 2011). These cannot be produced from the CB•drug complexes alone but several pharmaceutical adjuvants need to be incorporated in the formulation. Walker et al. reported a tablet formulation in which up to 50% microcrystalline CB6 (*w/w*) was mixed with other excipients such as lactose (as diluent/bulking agent), Avicel (aids tablet compaction), talc, magnesium stearate (as lubricants and glidants), and Ac-Di-Sol (as disintegrant) (Walker et al., 2010). The compatibility of CB6 with other excipients was confirmed by various techniques (Walker et al., 2010, 2011). The same group has successfully used CB6 in a nasal drug formulation containing hydroxypropyl methylcellulose (HPMC) and sodium carboxymethylcellulose (NaCMC) (Walker et al., 2011).

Use of CB7 in drug formulation was found to have the additional advantage that it prevents interconversion of crystal polymorphs of the drugs and allows them to retain the amorphous structure in the resulting CB7 complex (Jeon et al., 2005; Kennedy et al., 2009; Wheate et al., 2010; Saleh et al., 2012). It is also noteworthy that CB7 does not affect the surface area and pore size distribution which is beneficial for processing and robust formulation (Saleh et al., 2012).

In a very recent report, a “Trojan antibiotic” has been formulated by a host-guest complex of CB7 and a bola-type azobenzene compound with glycosylamine heads at both ends (Wang et al., 2019). Similar to the bacterial wall, this supramolecular assembly displays a surface that is fully decorated with sugar-like components. This Trojan antibiotic was found to be benign to a wide spectrum of bacteria at a weak basic pH of approximately 9.0 under daylight conditions but became a potent bactericide toward both Gram-negative and Gram-positive bacteria at pH 4.0 under 365 nm UV irradiation. The dual use of pH and UV light greatly enhanced the efficacy of the bactericidal effect such that the *MIC*_50_ value of the Trojan antibiotic was observed to be at least 10 times smaller than that of conventional drugs. The activity of the Trojan antibiotic automatically stopped upon removal of the UV source and reversal of pH which prevents the buildup of active antimicrobial materials in the environment. This novel approach may pave the way to a new era in the fight against bacterial resistance.

## Cucurbiturils in Photodynamic Therapy

CBs have also been explored in regard to their potential to serve as enhancement agents for photosensitizer drugs utilized in photodynamic therapy (PDT), which has recently been reviewed (Robinson-Duggon et al., 2018). PDT applications have been extended from discrete CB•photosensitizer host-guest complexes to elaborate nanomaterials and supramolecular assemblies. Wang and coworkers prepared, for example, CB6-based nano-capsules through direct alkylation of perhydroxycucurbit[6]uril with a ditopic linker. A photosensitive therapeutic payload, such as chlorin e6, was encapsulated within these nano-capsules for targeted PDT against cancer cells (Sun et al., 2019).

Another nanoscale CB-based PDT agent was constructed through a multi-step assembly by using a dipolar fluorescence compound (with carbazole as the electron-donor motif and pyridinium as the electron acceptor), CB8, and α-cyclodextrin-modified hyaluronic acid (HA-CD) (Wu et al., 2019). The carbazole fluorophore was a non-NIR emissive dye with an emission wavelength of 568 nm that was used as photosensitizer. Host–guest complexation with CB8 exhibited a marked red shift of the emission maxima of the dye to 662 nm, such that the binary assembly could not only be used as an efficient PDT agent but also as a targeted NIR lysosome imaging probe. When HA-CD was incorporated into the assembly, owing to the strong interactions between α-CD and the alkyl chain, the mixture resulted in a ternary nano-supramolecular assembly with targeting properties. In the presence of overexpressed acceptors on cancer cell surfaces, the assembly showed light toxicity toward cancer cells (A549) while the light cytotoxicity was found to be remarkably reduced for normal cells (293T). Thus, a complex system with an ability for NIR imaging and enhanced targeted PDT efficiency was successfully constructed using the orthogonal host–guest recognition with different macrocyclic molecules.

## Cucurbiturils for Alleviating and Modulating Side Effects of Drug Administration

CBs can also reduce the toxicity or mask other properties of encapsulated guest molecules. The reversal of the action of neuromuscular blocking agents is a prominent example in this line of successful applications of the action of CBs and their derivatives (Ma et al., 2012b). CB7 was found to reduce the cytotoxicity of polycations such as polyethylenimine or cationic dendrimers through complexation (Lim et al., 2002; Li et al., 2017; Huang et al., 2018c). At the same time, these systems were demonstrated to act as efficient gene carriers. It has also been demonstrated recently that CB7 complexation of paraquat (methyl viologen dichloride hydrate), a widely used herbicide, decreases under various conditions and effectively the toxicity *in vitro* and *in vivo* (Zhang et al., 2019b). In a recent report, CB7 was shown to inhibit seizures induced by small toxic molecules in both, zebrafish and mice models (Huang et al., 2018b), which has also been related to their complexation potential, which results in an effective detoxification. It has also been demonstrated that hexadimethrine bromide (HB), an agent which causes internal blood coagulation, can be efficiently captured by CB7 to control blood coagulation both *in vitro* and *in vivo* (Huang et al., 2018a). In another interesting application, it was found that CB7 is able to conceal the taste of the bitterest substance, denatonium benzoate (Yang et al., 2017).

A pH-induced toxicity switch has also been described by employing CB7 (Cheng et al., 2018). A triple-station guest (viologen-phenylene-imidazole or V–P–I) is used for CB7 complexation. The complex exhibits pH-directed translocation with high fatigue resistance (up to more than 100 cycles). Under basic pH, due to deprotonation of the imidazolium group (I station), CB7 positions itself around the viologen moiety (V station) and, thus, masks the toxicity of the viologen. Decreasing the pH into the acidic region protonates the imidazole group, affects a locomotion of CB7 to the phenylene (P) station, and thereby, the toxicity of the viologen unit becomes prominent. Cytotoxicity testing was performed *in vitro* on RAW 264.7 (murine macrophage) and BEL 7402 (human liver cancer) cell lines. It was observed that, in case of normal non-cancerous RAW 264.7 cells, there is significant masking of the toxicity when the guest is complexed within CB7. However, for cancerous BEL 7402 cells, no such difference could be observed. In RAW 264.7 cells CB7 is presumed to remain on station V since the local pH is ~7.4 while in the case of BEL 7402 cells, it shuttles to station P as their pH is significantly lower, around 6.8 (similar to the p*K*_a_ of the guest).

## Cucurbituril-Based Systems for Diagnostics and Other Biomedical Applications

Host-guest complexes of CBs have also been used for sensing, diagnostic, theranostic, and other relevant medicinal or bioanalytical applications. Monitoring enzymatic reactions by the tandem assay principle has been successfully implemented. The basic principle applied here is to form an inclusion complex of CB with an appropriate fluorescent dye whose affinity with CB lies ideally in between the binding constant of the substrate and the product of the enzymatic reaction of interest. For example, by using the Dapoxyl (a fluorescent dye)/CB7 reporter pair, the decarboxylation processes of different amino acids (Lys, Arg, His, Tyr, and Trp) to their corresponding biogenic amines (cadaverine, agmatine, histamine, tyramine, and tryptamine) can be conveniently monitored (Hennig et al., 2007; Nau et al., 2009). By using this principle along with the intrinsic enantiospecificity of decarboxylases for L-amino acid substrates, multi-parameter sensor arrays (for measuring concentrations of several amino acids in parallel) were designed that selectively signal the presence of a reactive pair of an L-amino acid and its corresponding decarboxylase (Bailey et al., 2008).

Numerous reports by Urbach and others have demonstrated binding of CBs to amino acids, peptides, proteins, biomolecules (e.g., neurotransmitters), and dyes, signifying applicability to extremely accurate biosensing applications at sub-nanomolar concentrations (Bush et al., 2005; Chinai et al., 2011; Smith et al., 2015). Indeed, sensors for various biomolecules using host-guest chemistry have been developed by several research groups (Biedermann et al., 2012a; Minami et al., 2012; Kasera et al., 2014; Sinn and Biedermann, 2018). The sequence-specific recognition property of CB7 can be transferred from sensing to separation applications. The groups of Urbach and Isaacs have, for example, coated mono-functionalized CB7 on a solid sepharose resin in order to separate proteins, namely human growth hormone as well as native insulin, in complex mixtures (Li et al., 2016c).

The binding pairs between CB7 and adamantyl- (AdA) or ferrocenyl-ammonium (FcA) were recently utilized by Kim as a supramolecular latching system for protein imaging, overcoming the limitations of protein-based binding pairs (Kim et al., 2018). Proteins in (or on) the cells were adamantylated/ferrocenylated using various labeling approaches. The strong affinity of AdA or FcA allows these proteins to latch to Cy3-CB7 which results in the successful visualization of the proteins with cells and *Caenorhabditis elegans*. Importantly, no interference from endogenous biomolecules was observed, enabling clear fluorescence images for accurate and precise analysis of protein locations using fluorescence microscopy.

**Figure 13:**
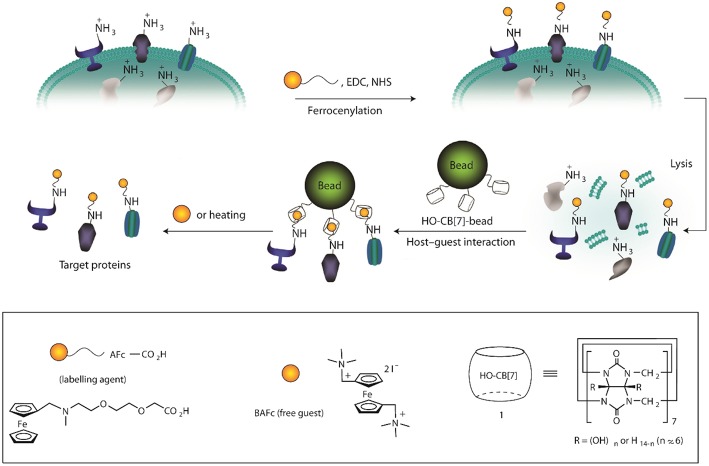
Schematic illustration for the isolation of plasma membrane proteins using a synthetic binding pair. EDC, 1-ethyl-3-(3-dimethylaminopropyl) carbodiimide; NHS, N-hydroxysuccimidyl sepharose (Lee et al., 2011) (Reproduced with permission, Copyright 2011, Nature Publishing Group).

Application of the sequence selectivity of CBs for aromatic peptides has been utilized to determine protease substrate selectivity and inhibition (Ghale et al., 2011). The selectivity of thermolysin to cleave the amide bond at the nitrogen side of Phe residues in peptides leads to the formation of peptide fragments with N-terminal Phe residues. The selectivity of CB7 toward N-terminal Phe residues has been used to create the assay. Scherman et al. have utilized this sequence selectivity of CBs to create a surface immobilized CB8 system which can be used to separate peptides with N-terminal tryptophan (Tian et al., 2011). Larger structures such as cells can also be adsorbed and released using surface-bound CB8 ternary complexes as shown by Sankaran et al. (2017). RGD-based tripeptide ligands were immobilized onto gold substrates fabricating an electrochemically controlled cell-adhesive surface (An et al., 2012). The RGD sequence can selectively adsorb cells on the surface. Electrochemical activation led to the dissociation of the host-guest complex and, thereby, the release of the adsorbed cells. A bio-interface has been developed by Kim and coworkers for isolating plasma membrane proteins by using highly selective binding of CB7 and a ferrocene derivative (AFc, Figure 13) (Lee et al., 2011). The system can capture model proteins from protein mixtures and the captured proteins were readily removed from the interface via addition of a second ferrocene derivative (BAFc) with higher binding affinity for CB7 than AFc. In a recent work, iron oxide nanoparticle surfaces were immobilized with CB7 and the modified particles were observed to be stable under a wide range of pH (2–12) (Benyettou et al., 2013). Nile red (NR) was loaded on the surface-bound CB7 and the nanoparticles were used for intracellular delivery of the dye and as MRI contrast agent demonstrating its potential for theranostics.

**Figure 14:**
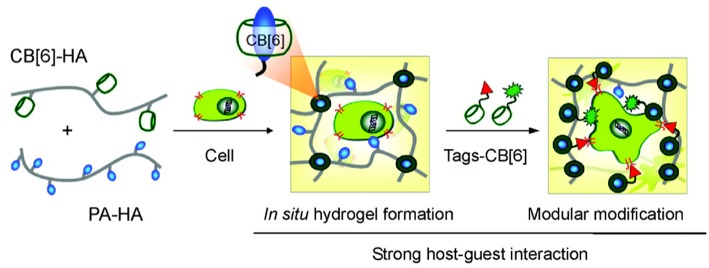
Schematic illustration for *in situ* formation of supramolecular biocompatible hydrogels (PA-HA, polyamine-hyaluronic acid assembly) and their modular modification using highly selective and strong supramolecular host-guest complexation (Park et al., 2012) (Reproduced with permission, Copyright 2013, American Chemical Society).

Tissue culture is another area where CBs have recently been successfully used. A facile *in situ* supramolecular assembly and modular modification of biocompatible hydrogels were demonstrated by Kim and coworkers (Park et al., 2012). CB6-conjugated hyaluronic acid (CB6-HA), diaminohexaneconjugated HA (DAH-HA), and tags-CB6 were used to create the hydrogel. When these hydrogels were modified with the c(RGDyK) peptide, the entrapped NHDF human fibroblast cells and NIH3T3 mouse fibroblast cells proliferated 5-fold within 14 and 3 days, respectively, compared to the untreated hydrogels (Figure 14). The 3D environment of the hydrogel was modularly modified by the simple treatment with various multifunctional tags-CB6. Furthermore, *in situ* formation of CB6/DAH-HA hydrogels under the skin of nude mice by sequential subcutaneous injections of CB6-HA and DAH-HA solutions was also confirmed. The fluorescence of modified FITC-CB6 in the hydrogels could be monitored for up to 11 days, showing the feasibility to deliver signals for cellular proliferation and differentiation in the body. To extend the work, the same group prepared 3D tissue-engineered supramolecular hydrogels using CB6-HA, DAH-HA, and drug-conjugated CB6 (drug-CB6) for the controlled chondrogenesis of human mesenchymal stem cells (hMSCs) (Jung et al., 2014). The system can be used as a platform for controlled drug delivery for cartilage regeneration and other various tissue-engineering applications.

In a recent study, Dowari et al. reported a three-way cross-linked peptide-based polymer (Dowari et al., 2018). The cross-linking was achieved *via* disulfide bond formation, enzymatic cross-linking by HRP-mediated dimerization of tyrosine, and supramolecular linkage using homoternary complexation by CB8. The supramolecular cross-linking was found to play a crucial role in controlling the size of the polymer. The surfaces of the polymer particles were decorated with an RGDS sequence which was utilized for efficient cell adhesion and proliferation of RAW 264.7 cells. The cross-linked polymers could bind cells effectively and the cells proliferated significantly. Jonkheijm and coworkers studied cell adhesion on multivalent knottins displaying RGD ligands with high affinity for integrin receptors (Sankaran et al., 2017). The integrin receptors were assembled on CB8/viologen-modified surfaces. The number of tryptophan units in the knottins varied between 0 and 4 which can form a heteroternary complex with CB8 and surface-tethered viologen. Specific binding occurred, and the affinity increased with the valency of the tryptophan residues on the knottin. Additionally, increased multilayer formation was observed, attributed to homoternary complex formation between tryptophan residues of different knottins and CB8. Control over the surface coverage of the knottins could thus be achieved by valency and concentration. Experiments with mouse myoblast (C2C12) cells on the self-assembled knottin surfaces showed specific integrin recognition by the RGD-displaying knottins. Cells were observed to elongate more on the knottin surfaces with higher valency. Moreover, more pronounced focal adhesion formation was observed on the higher-valency knottin surfaces.

## Toxicity and Permeability of Cucurbituril Macrocycles

Any potential biological or medicinal application depends on the cytotoxicity and biocompatibility of the employed formulations. In regard to CB-based compounds and nanomaterials, Kim and coworkers demonstrated the non-toxicity of CB molecules with ED_50_ levels of more than 100 μM against human lung and ovarian cancer cells (Jeon et al., 2005). *In vitro* cell viability testing of CB7 by using MTT assay in CHO-K1 cells showed no significant cytotoxicity up to 1 mM and 3 h incubation time; after 48 h incubation time an *IC*_50_ value of 0.53 mM was determined (Uzunova et al., 2010). Owing to the low solubility of CB8, a precise determination of its toxicity level is difficult. However, 20 μM CB8 caused a minor drop in cell viability (86%) within 48 h of incubation. A single oral dose of CB7 and CB8 as a mixture in equal proportions showed no toxicity up to 600 mg kg^−1^ (Uzunova et al., 2010). CB5, CB7, and several acyclic CB containers were also tested for their toxicology and bioactivity; they were found to be non-toxic within the desired concentration range (Hettiarachchi et al., 2010).

Another important question which needs to be addressed before using any system for biological application is their cell permeability. Acridine orange and pyronine Y complexes of CB7 and CB8 were employed to show that the complexes can penetrate the cell membrane of mouse embryo muscle cells (Montes-Navajas et al., 2009). CB7 complexes with dye molecules (fluorophores conjugated with spermidine and adamantylamine) were shown by Isaacs and coworkers to be able to cross the cell membranes of murine macrophage cells; within 20 min, 86% of the cells incorporated the complex. The host-guest complex was observed to be stable within the cells up to 2 h (Hettiarachchi et al., 2010). A CB7-labeled antibody has also been reported to be spontaneously taken up into living cells (Sasmal et al., 2018).

## Conclusions

We provided an overview of the recent achievements in the area of medicinal-chemical and chemical-biological applications utilizing the host-guest chemistry of CBs. Over the last decade, there has been a paradigm shift in the research with CBs and the focus is now more on the actual applications of these fascinating macrocyclic hosts. A major thrust is in the area of biological and specifically toward biomedical applications. CBs have been widely used to bind bioactive molecules, which helps to overcome the poor solubility of hydrophobic molecules, in particular drug candidates. The unique recognition properties and biocompatibility enables their implementation as excipients (Lim et al., 2002; Kuok et al., 2017). Recent achievements in the preparation of CB derivatives and analogs allow their incorporation into more intricate and applied research lines (Ayhan et al., 2015; Kim et al., 2018; Koc et al., 2018; Park et al., 2018; Sun et al., 2018; Zhang et al., 2018a, 2019a) in an effort to by-pass intrinsic limitations of the parent CBs, such as their stringent selectivity for guest binding and the low intrinsic solubility of CB6 and CB8. We contend that this account will provide a platform for understanding the potential of CBs toward applications in pharmaceutically and medicinally research and assist us in designing and creating new CB-based assemblies.

## Author Contributions

All authors contributed to the design and write-up of the review.

### Conflict of Interest Statement

The authors declare that the research was conducted in the absence of any commercial or financial relationships that could be construed as a potential conflict of interest.
